# Transport Mechanisms of 2D Nanoparticles across a Human Follicle-Associated Epithelium Model

**DOI:** 10.1021/acsomega.5c01703

**Published:** 2025-08-29

**Authors:** Sanoj Rejinold N, Ji-Yeong Kim, Geun-woo Jin, Goeun Choi, Jin-Ho Choy

**Affiliations:** † Intelligent Nanohybrid Materials Laboratory (INML), Department of Chemistry, College of Science and Technology, 65383 Dankook University, Cheonan 31116, Republic of Korea; ∥ Department of Chemistry and Nano Science, 26717 Ewha Womans University, Seoul 03760, Republic of Korea; ⊥ R&D Center, Hyundai Bioscience Co., Ltd., Seoul 07990, Republic of Korea; ∇ Department of Nanobiomedical Science, 65383 Dankook University, Cheonan 31116, Republic of Korea; ‡ Natural Sciences Division, National Academy of Sciences, Seoul 06579, Republic of Korea

## Abstract

Oral drug delivery is the most common route for drug administration, because of its safety and convenience. Although there have been many drug delivery systems (DDS), inorganic clay-based nanosystems have been less explored. In particular, zinc basic salt (ZBS) has never been used as an oral DDS though it has high biocompatibility, anionic exchange capacity, and controlled release behavior. Here, we demonstrated the potency of ZBS as an oral DDS via an in vitro human follicle-associated epithelium (FAE) model to observe transport pathways for various-sized 2D ZBS (200 and 1000 nm) across the cellular monolayers. The decrease in transepithelial electrical resistance (TEER) for the Caco-2 and Raji B coculture monolayers incubated with 2D nanoparticles suggests that the paracellular tight junctions became loosened, confirming that the present layered nanoparticles were interacting with the epithelial cell layer and could open the tight junctions to improve drug uptake via transcellular and paracellular transport pathways also confirmed by the confocal images of the coculture cell monolayer. It is also found that the 200 nm-sized ZBS particles showed better translocation than the 1000 nm particles without any toxicity. Therefore, the 200 nm ZBS nanoparticles could be suggested as a promising oral drug delivery carrier.

## Graphical abstract

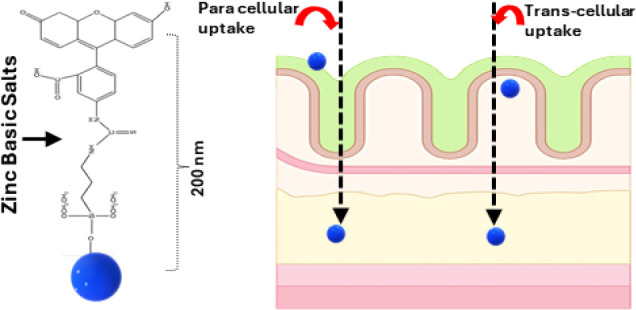

## 1 Introduction

Oral drug delivery is the most convenient and widely used route of administration, offering greater patient compliance compared to alternative methods such as intravenous, intraperitoneal, or transdermal delivery.^1–3^ 2D drug delivery carriers have gained immense attention in oral drug delivery research due to their unique physicochemical properties, including high surface area, tunable surface chemistry, and excellent mechanical stability. Lately, materials such as graphene oxide (GO)^4^ and molybdenum disulfide (MoS_2_)^5^ have demonstrated significant potential in drug delivery, bioimaging, and tissue engineering. GO, for instance, has been extensively studied for its ability to load and release therapeutic agents due to its ability be functionalized with abundant functional groups, while MoS_2_ exhibits excellent photothermal and photodynamic properties for cancer therapy.^5^

On the other hand, inorganic nanoparticles like zinc basic salts (ZBS) have a great attraction as oral drug delivery carriers due to their high biocompatibility, anionic exchange capacity, controlled release behavior, and controlled particle size with simple synthesis.^6–12^

ZBS has been suggested to encapsulate drugs into its interlayer space via intercalative ion-exchange reaction at a molecular level. When the hydrophobic drug molecules were intercalated into the ZBS lattice and further into the enteric coating, an enhanced solubility and sustained release of the drug could be realized resulting in improvement of absorption and bioavailability.^12–15^

Our previous studies on ZBS^11^ that encapsulated artesunate in ZBS at a molecular level showed maximum solubility and stability. Further enteric coating on ZBS hybrids can protect the sudden acidic responsive dissolution for ZBS, eventually benefiting improved oral absorption in a pharmacokinetic study. However, the mechanism behind such improved efficacy for ZBS hybrids was quite unknown. In this study, we investigated the cellular transport behaviors of ZBS through the epithelial barrier in view of the oral drug delivery system. We synthesized the two different sizes of ZBS particles, 200 and 1000 nm, and observed the transport via the in vitro FAE model. In particular, the transport behavior of 200 nm ZBS is a focal point, and the 1000 nm ZBS particle is a control group. In previous reported results,^16,17^ it was found that particles having a size range of 50 to 200 nm nanoparticles were selectively internalized into cells via clathrin-mediated endocytosis with improved permeability and retention, but the nanoparticles that are larger than 350 nm have no selectivity to cellular uptake.

As shown in Scheme 1A, intestinal villi are finger-like projections that extend into the lumen of the small intestine, greatly increasing the available surface area for material absorption. The mucosa, the innermost layer of the intestine, is composed of different cell types with a single layer and covered by mucus. The enterocytes, absorptive cells, are the most common epithelial cell type, and the second one is the mucus-secreting goblet cell. Moreover, the M cells, another important intestinal cell type, play a role in mediating the intestinal immune response. The gut-associated lymphoid tissue (GALT) is an important anatomical feature of the small intestine, and the epithelium overlying these dormal GALT structures is termed the follicle-associated epithelium.^18,19^ As illustrated in Scheme 1B, nanoparticles may cross the epithelial barrier via multiple pathways, including M-cell transcytosis, paracellular passage through tight junctions, and transcellular uptake into enterocytes. Based on this in vitro FAE model, which represents the intestinal epithelium model for oral drug delivery, one can evaluate the intracellular passage of the nanoparticles across the epithelial barrier. The in vitro FAE model differs from the normal intestinal epithelium such as the enterocyte-like model and mucus-secreting model, and it is based on coculture of human intestinal epithelial Caco-2 cells and human Raji B lymphocytes (Scheme 1C). The in vitro FAE model contains specialized epithelial microfold M cells with the capacity to transport an extensive range of materials, such as bacteria, viruses, macromolecules, and particles, and consequently the representative in vitro model mimicks the intestinal barrier for oral drug delivery.^19–24^

**Scheme 1:**
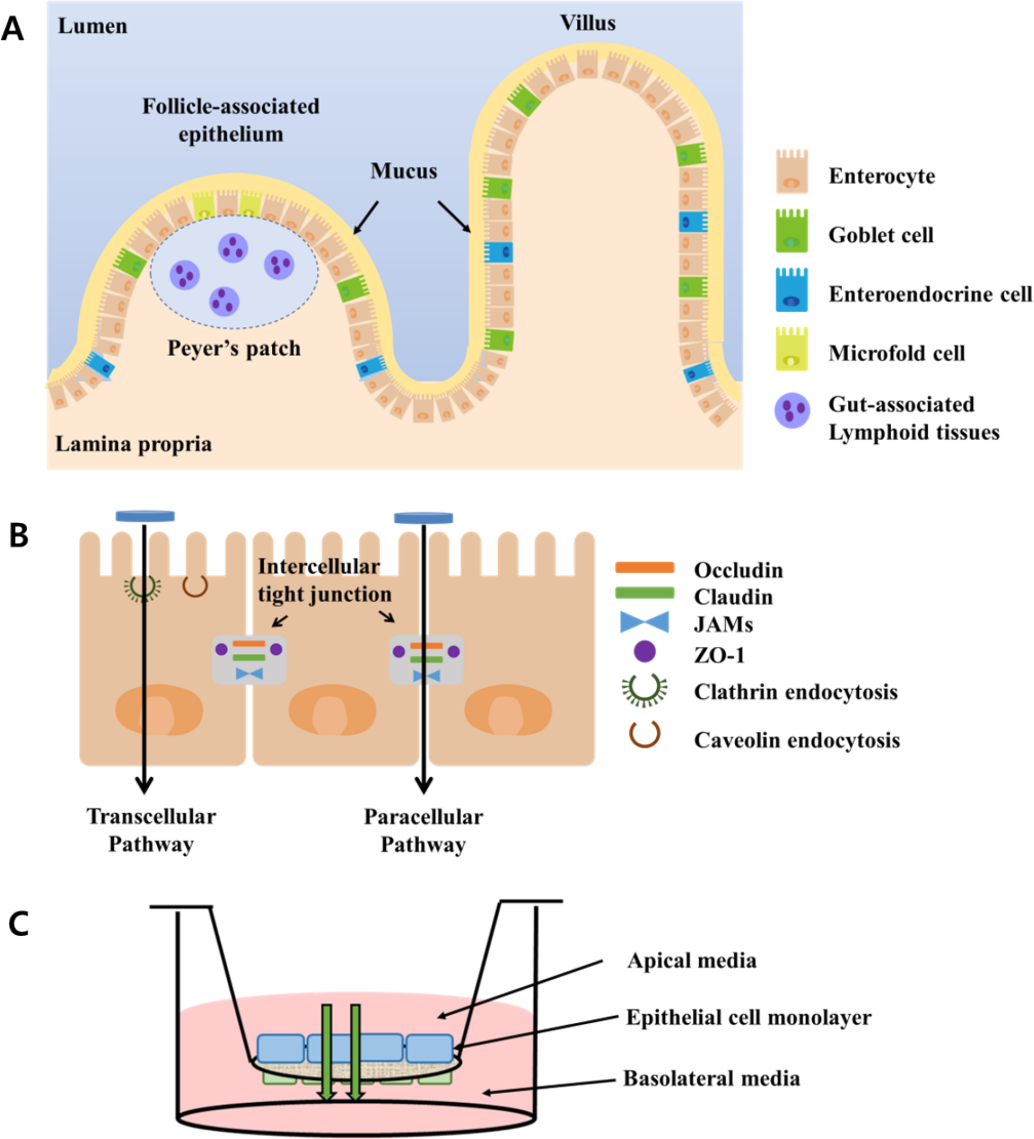
Proposed Transport Mechanisms of 2D ZBS Nanoparticles across the Intestinal Epithelium^a^

a (A) Schematic overview of the intestinal mucosa showing Peyer’s patch-associated follicle-associated epithelium (FAE) and villus structures. The epithelial layer is composed of different cell types, including goblet cells (mucus-secreting), enterocytes (absorptive cells), M cells (antigen-sampling), and immune cells in the lamina propria. Nanoparticles encounter the mucus barrier before interacting with epithelial cells. (B) Enlarged view of cellular transport routes through the epithelial monolayer. ZBS nanoparticles may cross the intestinal barrier via multiple mechanisms: M-cell-mediated transcytosis, wherein M cells facilitate uptake and transport of particulate antigens or nanosystems to underlying immune cells; paracellular transport, where smaller nanoparticles may diffuse through the tight junctions between epithelial cells; transcellular transport, involving endocytosis into enterocytes followed by intracellular trafficking and exocytosis toward the basolateral side. Receptor-mediated endocytosis, where surface-functionalized ZBS particles bind to specific cell surface receptors to trigger uptake. (C) Cross-sectional schematic of the in vitro FAE model, where a co-cultured epithelial monolayer (depicted as the blue color) consists of Caco-2 cells and Raji B cells to simulate M-cell differentiation. The 2D ZBS nanoparticles would be interacting with the epithelial barrier, with potential routes of transport including transcytosis via M cells, paracellular diffusion, and vesicular uptake by enterocytes. This model serves as a physiologically relevant platform to study nanoparticle absorption and barrier permeability under controlled conditions.

There are two ways to pass through the epithelial barrier, transcellular pathway and paracellular pathway, of which the former includes passive diffusion, active carrier mediated transportation such as clathrin and caveolin endocytosis, and transcytosis by M cell or enterocytes. On the other hand, the latter allows passage through the tight junction between the adjacent epithelial cells. Tight junctions are known to be regulated by proteins, which are junctional adhesion molecules (JAMs), occludins, and claudins (Scheme 1B).

There has been no investigation into the translocation of nanoparticles like ZBS across the intestinal epithelial cell in contrast with other inorganic,^25–29^ polymeric,^30–41^ and lipid nanoparticles.^42,43^ Polymer nanoparticles like chitosan and thiomers induce a reversible opening of the tight junctions due to their effect on depolymerization of cellular F-actin and on the tight-junction protein ZO-1,^19,29,31^ and also, other nanoparticles like silica and gold nanoparticles could possibly transport macromolecules (e.g., insulin, antigen, and protein) across the intestinal barrier as a physiochemical permeation enhancer.^20,21,25^ Functionalized nanoparticles (thiolated nanoparticle) with ligands for receptors expressed on the epithelial cell surface are transported by transcytosis.^37^ Also, particle size determines the way of cellular uptake in oral delivery, particles below 100 nm favor transcytosis, and particles larger than micro sized are trapped in Peyer’s patches, resulting in poor absorption. Moreover, very small particles (<50 nm) only allow for the paracellular transport.^13,15,19,44^ To directly observe the transport of ZBS on the epithelial barrier, transepithelial electrical resistance (TEER) values of the Caco-2 and Raji B coculture monolayers were measured with respect to the incubation time of 200 and 1000 nm ZBS nanoparticles. Also, the transport localization of cell monolayer-incubated 200 and 1000 nm ZBS was visualized using FITC-conjugated ZBS via confocal microscopy.

Therefore, the primary research questions addressed in this study are

1) How do 2D ZBS nanoparticles of different sizes traverse epithelial cell barriers?
2) What are the potential mechanisms facilitating their transport?

We hypothesize that smaller 2D ZBS nanoparticles (∼200 nm) can penetrate epithelial cells predominantly through trans- and paracellular pathways. These findings could position ZBS-based 2D inorganic nanomaterials as promising candidates for the development of oral drug delivery systems.

## 2 Materials and Methods

### 2.1 Synthesis and Characterization of the Nanoparticle

200 and 1000 nm-sized ZBS were prepared by the coprecipitation method. The 1000 nm-sized ZBS was prepared in decarbonated water, where as the 200 nm ZBS was prepared in 75% ethanol solution. The ZnCl_2_ dissolved in decarbonated water or 75% ethanol, and then, each ZnCl_2_ solution was titrated with NaOH at pH 7 under a nitrogen atmosphere to prevent carbonate contamination from air. The reaction solution was stirred for 24 h. Then, the white-colored suspension was centrifugated to separate ZBS nanoparticles from solution (14,000 rpm, 10 min), and then the prepared precipitate was washed three times with decarbonated water to avoid carbon contamination. Finally, the product was freeze-dried.

For conjugation of fluorescein 5-isothiocyanate (FITC) on the ZBS surface, each 200 and 1000 nm ZBS nanoparticles were dispersed in ethanol and 3-aminopropyltriethoxysilane (APS) dropwise. The reaction solution was stirred for 24 h at room temperature, and then the white suspension was washed and freeze-dried. Then, the silylated ZBS (ZBS-APS) was dispersed in deionized water, and the FITC solution was mixed in ethanol. The reaction solution stirred for 12 h at room temperature, and then a yellow-colored suspension was washed and freeze-dried. The chemical formulas for all the samples are given in Table S1.

For structural analysis, the powder X-ray diffraction (PXRD) patterns were collected using a diffractometer (D/Max-2200, Ultima, Rigaku, Japan) with Ni-filtered Cu Kα radiation (λ = 1.5418 Å) at 40 kV and 30 mA. Fourier transform–infrared (FT-IR) spectra were obtained using a JASCO FT-IR 6100 spectrometer (JASCO, Japan) by the standard KBr disk method. The chemical analysis for all the samples was made by an ICP-Optical Emission Spectrometer (Optima 8300, PerkinElmer). The morphology for the diverse nanoparticles was studied with a field emission scanning electron microscopic image (FE-SEM; JEOL JSM-6700F; Japan). ^29^Si CPMAS NMR data were acquired on a 400 MHz solid-state NMR spectrometer (AVANCE III HD, Bruker, Germany) at the KBSI Western Seoul center.

### 2.2 Cell Culture

Human colon adenocarcinoma Caco-2 line and CCD-18Co colon normal cell line were purchased from the Korean Cell Line Bank. The human Burkitt’s lymphoma Raji B cell line was purchased from ATCC. Minimum essential media (MEM), Dulbecco’s Modified Eagle Media (DMEM), RPMI 1640 media, and Hank’s Balanced Salt Solution buffer (HBSS) were purchased from Gibco (Korea). Caco-2 cell, CCD-18Co, and Raji B cell were cultured in MEM and RPMI 1640 media, respectively, supplemented with 10% fetal bovine serum and 1% penicillin–streptomycin under 5% CO_2_ water-saturated atmosphere at 37 °C.

### 2.3 Cytotoxicity of Nanoparticles

The cytotoxicity of nanoparticles was investigated by the MTT (2-(4,5-dimethylthiazol-2-yl)-2,5diphenyltetrazolium bromide) assay. CCD-18Co colon normal cells were seeded in 96-well plates at a density of 5 × 103 cell/well and then exposed to 50–300 μg/mL particles for 4 h. Furthermore, MTT solution was added to each well, which was then incubated for 4 h. The MTT medium was removed and replaced with DMSO to mix with formazan crystals. The absorbance at 540 nm was measured with an ELISA reader. Raji and Caco-2 cells were used to evaluate the effects of the ZBS particles on cell viability. Both cell types were cultured in RPMI 1640 medium supplemented with 10% fetal bovine serum (FBS) and 1% penicillin–streptomycin, maintained in a humidified incubator at 37 °C with 5% CO_2_.

For Caco-2 cells, once they reached approximately 80% confluency in a 175 T flask, they were trypsinized using 0.25% trypsin-EDTA containing phenol red. The trypsin was neutralized with 10 mL of complete medium, and the suspension was transferred into a 50 mL conical tube, followed by centrifugation at 1000 rpm (approximately 220 × *g*) for 5 min at 4 °C using a refrigerated centrifuge. After the supernatant was discarded, the cell pellet was resuspended in fresh medium and counted using an automated cell counter. Cells were then seeded at a density of 5 × 10^3^ cells per well in white 96-well plates (Corning #3610), observed under a microscope for uniform distribution, and incubated for 24 h to allow stabilization.

Raji cells were gently homogenized with a pipet, transferred into a conical tube, and centrifuged under the same conditions. After the supernatant was discarded, cells were resuspended in fresh medium and counted. They were seeded into 96-well plates at a concentration of 5 × 10^3^ cells per well. Following microscopic confirmation of cell distribution, the plates were incubated for 4 h under standard conditions to stabilize the cells.

The test substances used in this study were two different sizes of ZBS (zinc borosilicate) particles. Sample 1 consisted of 200 nm ZBS particles, and sample 2 contained 1000 nm ZBS particles. Both were provided in powder form and dissolved in complete medium to prepare 200× stock solutions. These were diluted to the desired final treatment concentrations and added to 96-well plates containing cells. After treatment, plates were incubated at 37 °C in a 5% CO_2_ incubator for 4 h.

Cell viability was assessed using the CellTiter-Glo luminescent assay (Promega). The reagent was prepared freshly by mixing the buffer and substrate in a 1:1 ratio at room temperature. 100 μL of the reagent was added to each well containing 100 μL of cell suspension. Plates were kept at room temperature for 10 min to induce cell lysis and allow luminescence stabilization. Luminescence (RLU) was measured by using a BioTek Synergy H1 microplate reader. Liquid handling steps were performed using ep Dualfilter T.I.P.S. pipet tips (1–100, 2–200, and 50–1000 μL, Eppendorf), and microcentrifuge tubes (1.5 mL) were sourced from GeneAll.

RLU data were extracted from Gen5 software and analyzed in Excel. Values were normalized against those of the control group to calculate relative viability percentages. Statistical processing and data visualization were also carried out using Excel.

All cell work was conducted in a Class II biological safety cabinet (Thermo Fisher 1300 series). Incubation was performed using a Thermo Fisher 3311E incubator. Cell morphology was checked using a ZEISS First Vert microscope, and cell counts were measured using a Countess 3 FL cell counter from Thermo Fisher. Standard laboratory reagents like ethanol and DMSO (both from Sigma-Aldrich) were used where required throughout the experiment.

### 2.4 Nanoparticle Transport

In vitro FAE model was prepared according to the protocol developed by Rieux et al.^39^ In addition, there were many reports on FAE model-based nanoparticle uptake.^45,46^ Transwell polycarbonate inserts were coated with a Matrigel basement membrane matrix. Caco-2 cells (5 × 10^5^ cells) were seeded in the upper side and incubated for 14 days. Then, Raji B cells (5 × 10^5^ cells) were added to the basolateral insert compartment, and the cocultures were maintained for 5 days. Each nanoparticle was dispersed in Hank’s balanced salt solution (HBBS) at 250 μg/mL concentration. The apical media were replaced by a nanoparticle solution and incubated. TEER of the cell monolayers was measured before and after the nanoparticle transport experiments by means of a Millicell ERS-2 (Millipore Corporation, Bedford). Each TEER value was calculated as a percentage of initial TEER values.

### 2.5 Localization of Nanoparticles in the Cell Layer

After the transport experiment, inserts were washed with PBS and fixed in 4% formaldehyde. Actin was stained with 250 μL of rhodamine-phalloidine (4 U/mL) in HBSS with 0.2% Triton X-100 for 10 min to reveal cell borders. Inserts were washed in HBSS, cut, and mounted on glass slides. Cell monolayers and nanoparticles were observed with confocal microscopy.

### 2.6 Quantification of Fluorescence

Fluorescent images were quantified by using ImageJ software (NIH, USA). The images were converted to grayscale (8-bit), and thresholding was applied to distinguish the fluorescence signal from the background. Regions of interest (ROIs) were selected, measurements were set, and quantification was performed. The results were then saved for further analysis.

## 3 Results and Discussion

### 3.1 Nanoparticle Characterization

ZBS with two different particle sizes were prepared by the coprecipitation method; there are several factors affecting particle size, which are temperature, aging time, and concentrations of metal ion and solvent.

As previously shown by Choy et al., the particle size of layered double hydroxide (LDH) nanostructures affects their biological behavior. Zinc basic salts (ZBS), though not true LDHs, possess similar layered frameworks and show comparable size-dependent transport and interactions, which are significantly influenced by both aging time and reaction temperature.^47^ An extended aging period or increased synthesis temperature typically facilitates crystal growth, leading to the formation of larger particles. However, in the case of zinc-containing systems, temperature modulation poses a specific limitation. Elevating the reaction temperature above 40 °C promotes the formation of zinc oxide (ZnO) as an unwanted byproduct alongside the desired ZBS phase, as confirmed by the structural features observed in Figure S1. This phase competition compromises the purity and uniformity of the ZBS nanostructure.

To overcome this challenge and suppress ZnO formation, the reaction parameters were modified, specifically the solvent system and ethanol content. Instead of relying on thermal control, we employed a solvent-mediated size tuning approach to regulate ZBS particle dimensions while maintaining phase purity. Ethanol was used as a cosolvent to influence the supersaturation and nucleation kinetics, effectively tailoring the particle size. When 75% ethanol was introduced into the system, it facilitated the synthesis of ZBS nanoparticles with a mean diameter of ∼200 nm, likely due to the reduced dielectric constant and altered solvation dynamics, which limit excessive crystal growth (Figure S2). In contrast, ZBS particles synthesized in decarbonated water under identical molar ratios and reaction conditions yielded particles with an average size of ∼1000 nm, indicating the importance of the solvent environment in modulating nucleation rates and particle aggregation.

The surface morphology and size distributions of both formulations were further validated through scanning electron microscopy (SEM). As shown in Figure 1A, SEM micrographs reveal that the 200 nm ZBS particles (Figure 1A-a) and 1000 nm ZBS particles (Figure 1A-b) both possess a characteristic plate-like morphology, consistent with LDH type materials but differ significantly in scale. This particle size control is critical for tuning cellular uptake, in vivo distribution, and biological interactions in downstream biomedical applications.

**Figure 1:**
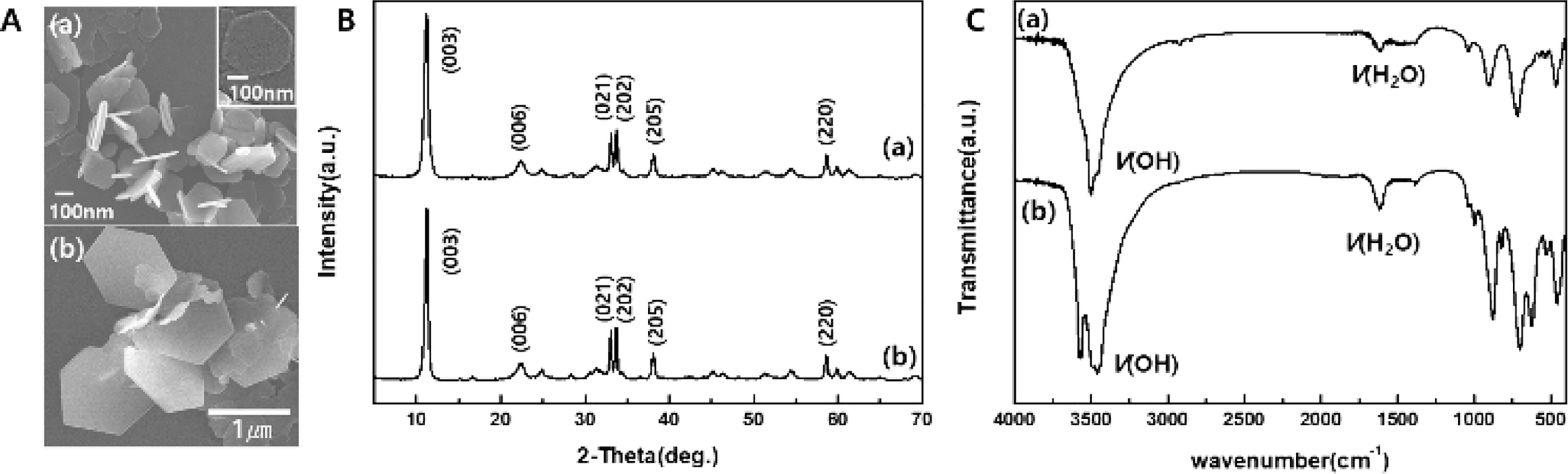
(A) SEM images of (a) 200 nm and (b) 1000 nm ZBS nanoparticles. (B) PXRD patterns of (a) 200 nm and (b) 1000 nm ZBS nanoparticles. (C) FT-IR spectra of (a) 200 nm and (b) 1000 nm ZBS nanoparticles.

The pristine ZBS nanoparticles (200 and 1000 nm) exhibited moderately positive surface charges with zeta potentials of 19.4 ± 1.5 and 21.2 ± 1.6 mV, respectively. These values are attributed to the presence of hydroxyl and surface zinc ions exposed in the platelet-like lamellar structure of Zn_5_(OH)_8_Cl_2_·*x*H_2_O. The slight increase in zeta potential with increasing particle size may be associated with variations in hydration layers and surface exposure of active hydroxyl groups.

Upon surface functionalization with APS and FITC, both formulations displayed a modest increase in zeta potential, indicating successful grafting of APS moieties and FITC conjugates. The presence of positively charged amine groups on APS contributes to this enhanced surface potential. The degree of modification is reflected in the appended mole fractions: 2.43 APS and 0.051 FITC per formula unit for 200 nm ZBS, and slightly higher incorporation (2.73 APS and 0.057 FITC) for 1000 nm ZBS, possibly due to increased surface area and porosity in the larger particle structure. These modifications not only preserve the colloidal stability of the ZBS nanoparticles in physiological environments but also provide functional handles for subsequent targeting or imaging applications, critical for cell uptake and gastro intestinal absorption studies.

Figure 1B shows the PXRD patterns of (a) 200 nm ZBS and (b) 1000 nm ZBS. In the diffraction pattern of 200 and 1000 nm size ZBS, the *d*-spacing at 11.3° indicates 7.86 Å corresponding to the (003) peak, which indicates that the basal spacing of Cl– is incorporated in hydroxide layers. Figure 1C shows the FT-IR spectra of (a) 200 nm ZBS and (b) 1000 nm ZBS. For ZBS, the H_2_O bending vibration band and the O–H stretching vibration band are shown at 1640 and 3480 cm^–1^, respectively. The lattice vibrations of the ZBS sheets are observed by the occurrence of O–Zn–O at 470 cm^–1^ and Zn–O at 850 to 600 cm^–1^.

In the diffraction pattern of 200 nm size and 1000 nm size ZBS, the *d*-spacing at 11.3° indicates 7.86 Å corresponding to the (003) peak, which indicates that the basal spacing of Cl– is incorporated in hydroxide layers. Figure 1C shows the FT-IR spectra of (a) 200 nm ZBS and (b) 1000 nm ZBS. For ZBS, the H_2_O bending vibration and O–H stretching vibration bands are shown at 1640 and 3480 cm^–1^, respectively. The lattice vibrations of the ZBS sheets are observed by O–Zn–O at 470 cm^–1^ and Zn–O at 850 to 600 cm^–1^.

For effective tracking of pathways through the cell monolayer, ZBS nanoparticles were labeled with FITC. The covalent bonding between FITC and ZBS was realized by a silane coupling reaction and thiourea bond formation, as shown in Figure 2A. The surface hydroxyl groups of ZBS were modified by APS, and then the thiourea bonds were formed by covalent conjugation between the amine terminal of APS and the isothiocyanate terminal of FITC. The grafting of APS and subsequent conjugation of FITC molecules to ZBS nanoparticles was verified by PXRD patterns, FT-IR spectroscopy, ^29^Si CP MAS NMR, and UV–vis spectroscopy (Figures 2B,C and 3 and Figures S3–S5).

**Figure 2:**
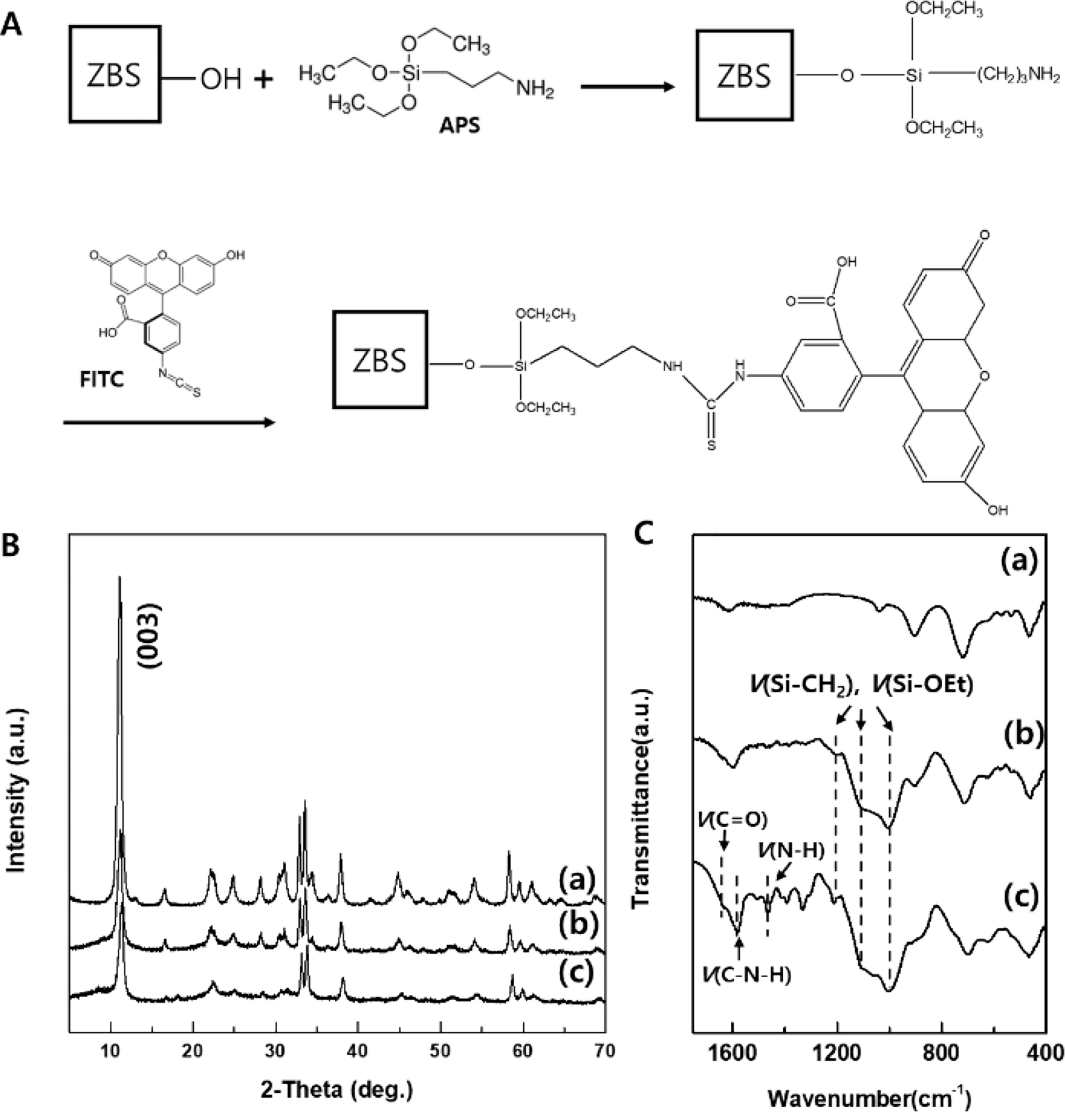
(A) Reaction scheme of APS grafting and subsequent FITC conjugation on the ZBS surface. (B) PXRD patterns of (a) 200 nm ZBS, (b) ZBS-APS, and (c) ZBS-APS-FITC. (C) FT-IR spectra of (a) 200 nm ZBS, (b) ZBS-APS, and (c) ZBS-APS-FITC.

**Figure 3:**
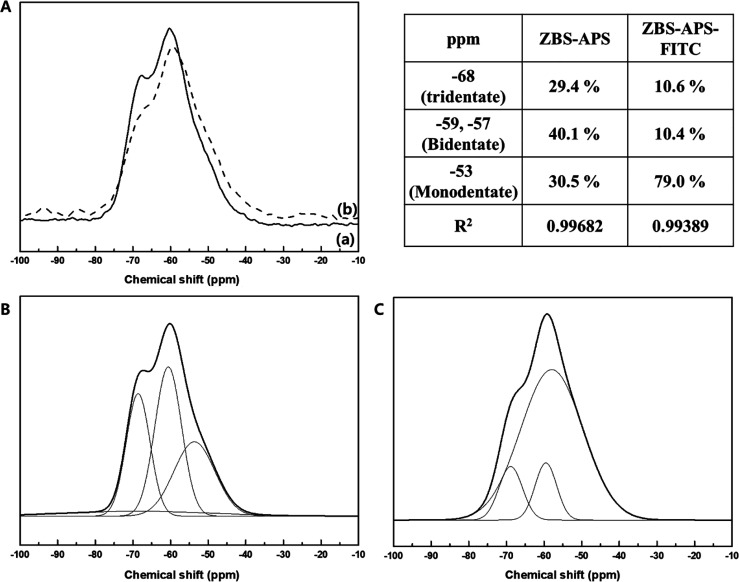
(A) ^29^Si CP MAS NMR spectra (a) ZBS-APS and (b) ZBS-APS-FITC. Deconvolution analyses of (B) ZBS-APS and (C) ZBS-APS-FITC.

The PXRD patterns of ZBS for each conjugation stage revealed that the crystal structure of ZBS was not influenced by the grafting reaction, as shown in Figure 2B and Figure S3a. Figure 2C and Figure S4 show the FT-IR spectra of (a) 200 nm ZBS, (b) ZBS-APS, and (c) ZBS-APS-FITC. For ZBS-APS, the distinct peaks at 1198, 1090, and 1001 cm^–1^ corresponding to v­(Si-CH_2_) and v­(Si-OEt) are observed in APS grafting.^12,30,31^ For ZBS-APS-FITC, the characteristic bands at 1630, 1459, and 1580 cm^–1^ are assigned to C=O stretching vibration, N–H stretching vibration, and C–N–H stretching vibration.

From the ^29^Si CP MAS NMR result as shown in Figure 3, we confirmed that silane moieties are covalently bound to the ZBS surface and covalent bonds are maintained after FITC conjugation. The broad NMR peak of ZBS-APS at around −59 ppm was decomposed to three peaks at −68, −59, and −53 ppm, which correspond to tridentate (T3), bidentate (T2), and monodentate (T1) siloxane bonds, respectively. The relative percentage of each oxane bond is obtained by integrating the peak area, showing 29.4, 40.1, and 30.5% of relative population for the T3, T2, and T1 bonds, respectively. Therefore, the covalent siloxane bonds are strongly formed between the APS molecules and ZBS surface.^48–50^ Also, the broad NMR peak of ZBS-APS-FITC at around −59 ppm is decomposed to three peaks at −68, −57, and −53 ppm, which correspond to T3, T2, and T1 siloxane bonds, respectively. The relative percentage of each oxane bond is also gained by integrating the peak area, showing 10.6, 10.4, and 79.0% of relative population for T3, T2, and T1 bonds, respectively. Therefore, it is concluded that the siloxane bonds are strongly bound to the ZBS surface after FITC conjugation.

### 3.2 Nanoparticle Transport

To evaluate the toxicity of ZBS nanoparticles to particle size, cell proliferation was measured with an MTT assay in CCD-18Co colon normal cells, as shown in Figure 4A. The MTT assay was performed during 4 h of incubation of nanoparticles for equal condition of the transport experiment. The cell viability of the 1000 nm ZBS nanoparticle for 4 h was 78.2%, which is a lower cell viability than that of 200 nm ZBS (94%) due to its size.^51–53^

**Figure 4:**
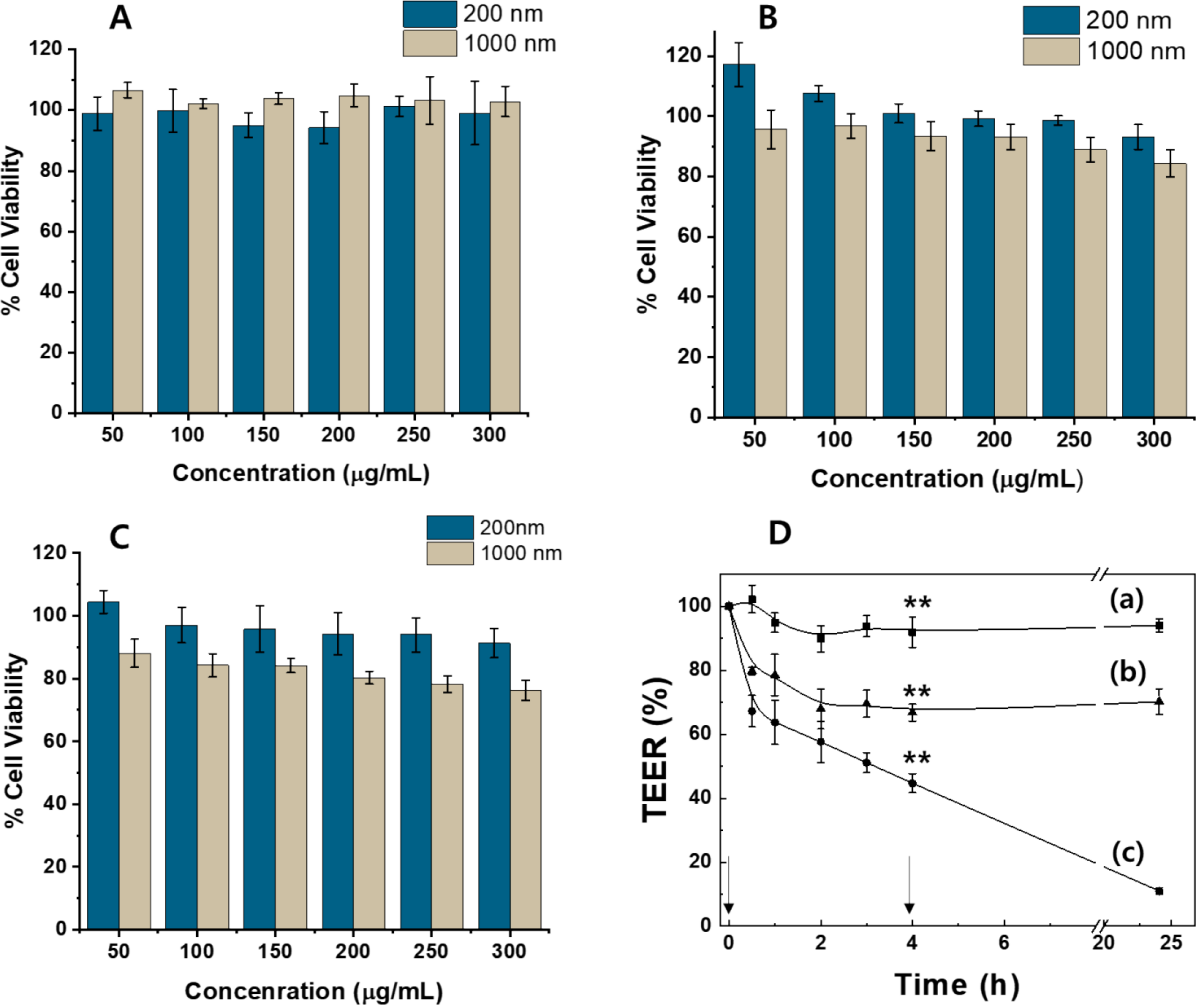
Cell viability of 200 and 1000 nm ZBS on (A) Caco-2, (B) Raji B, and (C) CCD-18Co colon normal cell line cells for 4 h. (D) TEER values of 200 nm ZBS (b) and 1000 nm ZBS (c); ((a) control group). Statistical analysis was performed with a *p*-value <0.05 (*) or <0.01 (**).

The intracellular transport behaviors of ZBS through the in vitro FAE model were investigated with TEER measurement and confocal microscopy. As mentioned above, the epithelial cell layer maintains epithelial integrity, which reflects the tight junction between the adjacent cells. The integrity of Caco-2 and Raji B coculture monolayers was checked by determining their TEER value. The average TEER value of the control group was determined to be 545 ± 24 Ω·cm^2^, which indicates that Caco-2 and Raji B coculture monolayers were developed well.

Figure 4D shows the TEER values upon the addition of the two types of ZBS nanoparticles with respect to time. To evaluate the effect of the particle size on transport, nanoparticles were added at the apical side. All nanoparticles were incubated for 4 h and after withdrawal in the apical side (arrow). By quantitatively measuring the TEER value of the monolayer, it is possible to monitor the disruption of tight junctions by nanoparticles. When nanoparticles loosen the tight junction and interfere with the integrity of the epithelial monolayer, the TEER values will be reduced and the magnitude of TEER reduction will reflect the amount of damage to the monolayer. The TEER values of all ZBS nanoparticles dropped sharply within 30 min and then continued to decrease for 4 h. For 200 nm ZBS, the TEER value shows an about 30% decrease. The drop in the TEER indicates that the ZBS nanoparticles loosen the tight junctions connecting the epithelial cell, allowing nanoparticles to pass through the monolayer via the paracellular route. After withdrawal of all the nanoparticles incubated for 4 h, the TEER values recovered to above 70% of the initial value, which indicates the maintenance of the integrity of the cell monolayer, except for 1000 nm ZBS. However, in the case of 1000 nm ZBS, the TEER value shows the 55% decrease within 4 h. Moreover, after removal of the nanoparticle, the TEER value did not recover but reduced to 11% of the initial value. TEER values also indicate the toxic effects induced by nanoparticles, as damage to the tight junctions would allow the leakage of potentially toxic substances through the epithelial barriers into circulation.^22^ These results are in agreement with earlier observations, indicating that reductions in particle size are associated with more pronounced decreases in TEER values. Whitehead et al. suggested that the particle size inversely correlated with epithelial permeability for below 200 nm of silica nanoparticle.^26^ As illustrated by Rieux et al., 0.2 μm latex particles were more transported than 0.5 μm latex particles.^40^ Also, Zhang et al. showed that the adsorption rates were greater for large hematite nanoparticles (76 and 98 nm) than those for small hematite nanoparticles (26 and 53 nm).^29^ Also, Kadiyala et al. showed that the transcytosis efficiency was dropped in increasing the size from 100 to 500 nm of chitosan nanoparticles.^41^ Figure 5 shows the confocal microscopy images of the cell monolayer after incubation with ZBS nanoparticles. Actin (cell membrane) was stained with rhodamine-phalloidin (red), and ZBS nanoparticles were labeled with FITC (green). As shown in Figure 5, some of the green-labeled ZBS nanoparticles are internalized by the cell and other ZBS nanoparticles overlap with the cell membrane (red), which indicates that ZBS nanoparticles interacted with the monolayers and affected both transcellular and paracellular transport.

**Figure 5:**
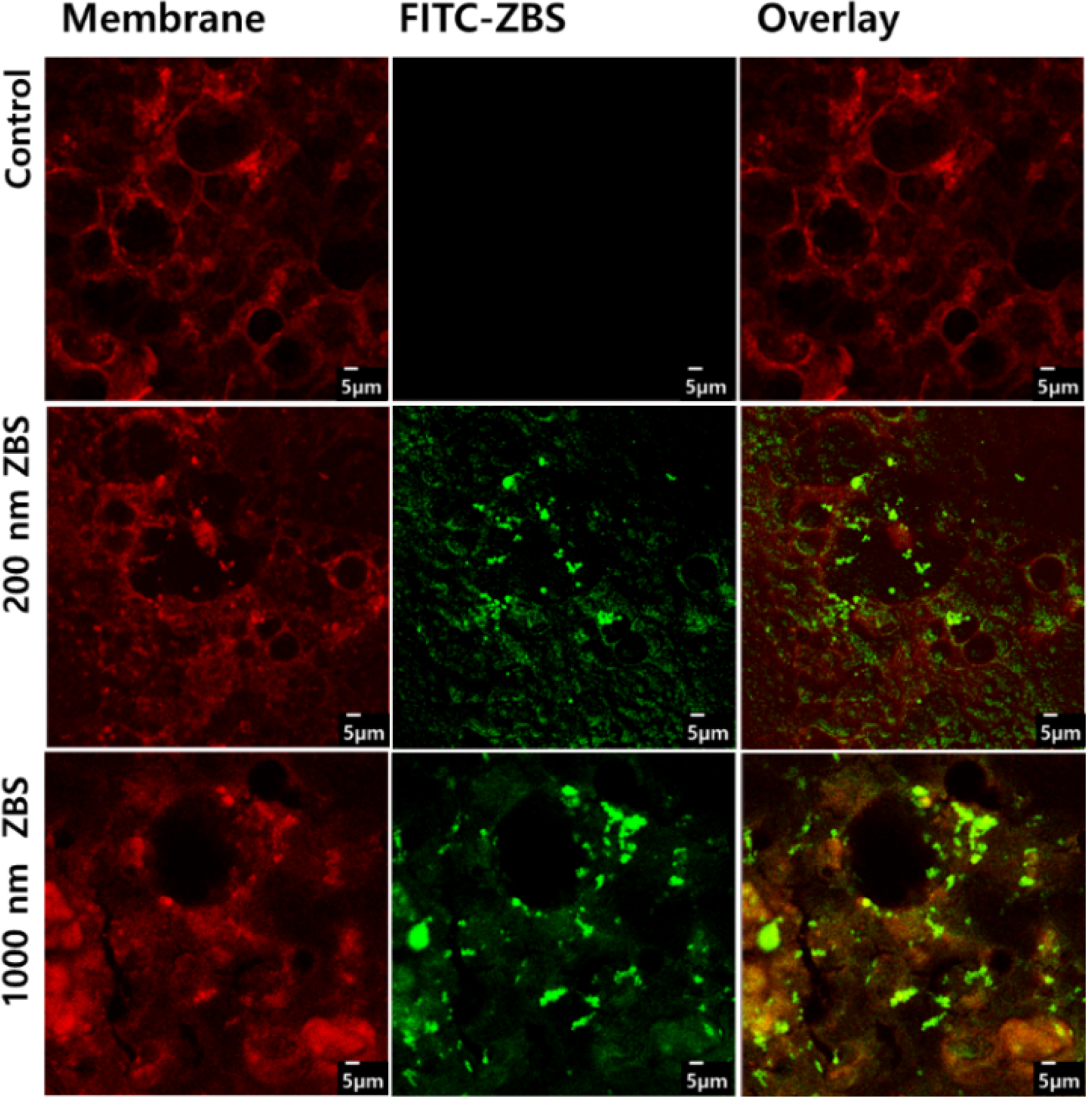
Confocal laser scanning microscopy (CLSM) images showing *x*-plane views of Caco-2 monolayers following incubation with fluorescently labeled ZBS-APS-FITC nanoparticles of different sizes. Red fluorescence indicates the cellular actin cytoskeleton stained with phalloidin–TRITC, while green fluorescence corresponds to the internalized ZBS-APS-FITC nanoparticles. The merged images demonstrate nanoparticle uptake and distribution within the cell layer. The panels represent the following: top row—control Caco-2 cells without nanoparticle exposure; middle row—cells treated with 200 nm ZBS-APS-FITC nanoparticles; bottom row—cells treated with 1000 nm ZBS-APS-FITC nanoparticles; scale bar = 5 μm.

In general, the in vitro FAE model mimics the intestinal barrier and allows evaluation of nanoparticle passage across the epithelial layer. According to other studies of transport behavior of nanoparticles across the epithelial barrier,^20,21,20,21,25–27,44^ small (<100–200 nm) and positively charged nanoparticles interacted more with the epithelial layer and acted as a permeation enhancer that opens the tight junctions to enhance oral drug uptake. ZBS nanoparticles, anionic clay, have a positive surface charge (Table S1) and have potential as a permeation enhancer of the epithelial barrier. It has already been proven that positively charged particles were more absorbed than the neutral or negative ones.^44^ Taken together, the enhanced oral absorption observed for artesunate encapsulated in ZBS, relative to intact artesunate in our earlier pharmacokinetic study, can be attributed to the selective intestial permeation of 200 nm ZBS nanoparticles.^11^ To summarize the results, 200 nm ZBS nanoparticles can open the tight junctions to improve intestinal permeability via the transcellular and paracellular pathways without any toxicity, different with 1000 nm ZBS nanoparticles, also confirmed by the TEER values and the confocal images of the coculture cell monolayer. Therefore, the 200 nm ZBS nanoparticles are suitable as an oral drug delivery carrier.

The cellular uptake and permeation of the nanoparticles are strongly influenced by their size and surface chemistry. In our study, ZBS-APS-FITC nanoparticles exhibited distinct permeation patterns between the 200 and 1000 nm formulations, as determined by transepithelial electrical resistance (TEER) measurements. Interestingly, while TEER data suggested higher permeability for the smaller (200 nm) particles, confocal laser scanning microscopy (CLSM) and fluorescence quantification (Figure S6) showed greater fluorescence intensity associated with the 1000 nm particles. This apparent contradiction may be attributed to larger particles aggregating at the cell surface or becoming trapped at the membrane interface. As a result, they exhibit increased surface-associated fluorescence but limited internalization, which aligns with the observed lower TEER reduction, indicating reduced transcellular transport compared to that of the smaller nanoparticles.

Interestingly, when comparing this permeation behavior to graphene oxide (GO) and its derivatives—such as reduced graphene oxide (rGO)—important parallels and differences arise. Previous studies, including those on rGO-exposed A549 cells, have shown that nanosheet-like graphene materials also undergo endocytosis, localizing primarily within late endosomes or lysosomes, like our findings with FITC-labeled ZBS nanoparticles.^54^ However, rGO exposure, especially at higher concentrations (>10 μg/mL), is associated with cytotoxicity, EMT induction, and pulmonary fibrotic responses, whereas the ZBS-APS-FITC hybrids were well tolerated in both Caco-2 and Raji B cell lines even at higher concentration ranges from 50 to 300 μg/mL, as demonstrated in our viability assays (Figure 4A–C).

A key distinction lies in the biological response post-permeation. While rGO disrupts cellular homeostasis, modulating key EMT markers (e.g., decreased E-cadherin, increased vimentin, VEGF-B, and TWIST1), the ZBS-APS-FITC particles did not induce any cytotoxicity, suggesting a safer interaction profile.

These findings emphasize that the particle size and dispersion stability play a central role in determining cellular interaction outcomes. While both ZBS nanoparticles and GO enter cells via endocytosis, only the latter shows deleterious biological reprogramming, possibly due to its 2D sheet structure, surface defects, and oxidative properties.

Therefore, ZBS-APS-FITC 200 nm particles demonstrate superior and safer permeation compared to their 1000 nm counterparts and offer a profile contrasting with that of graphene oxide, which, though permeable, poses cytotoxic and fibrogenic risks. This suggests a potential biomedical advantage of optimized nano-ZBS over conventional nanocarbons for epithelial barrier models or oral delivery applications.

## Perspective

The findings of this study highlight the potential of 2D zinc basic salts (ZBS) as promising nanocarriers for oral drug delivery, offering a unique alternative to conventional polymeric and lipid-based systems. By demonstrating the ability of ZBS nanoparticles to modulate epithelial permeability and enhance translocation across the FAE, this study opens new avenues for the rational design of inorganic nanocarriers with tunable transport properties. The observed size-dependent transport efficiency, with 200 nm ZBS exhibiting superior permeability without cytotoxic effects, underscores the importance of optimizing nanoparticle dimensions for enhanced bioavailability.

Looking ahead, future research will focus on translating these in vitro findings into preclinical and clinical settings to validate the efficacy and safety of ZBS-based oral drug delivery systems. Investigating the interactions of ZBS nanoparticles with mucosal immunity, their stability in the gastrointestinal environment, and their long-term pharmacokinetic behavior will be critical for advancing their application. Additionally, leveraging surface modifications, ligand conjugation, or hybridization with biomolecules could further enhance targeted delivery and controlled release.

This work serves as a foundation for expanding the role of inorganic nanocarriers in oral therapeutics, with ZBS emerging as a versatile platform for improving drug absorption and therapeutic outcomes. By bridging nanotechnology and mucosal drug delivery, this study paves the way for next-generation oral nanomedicines that could revolutionize patient care and treatment strategies.

## Conclusions

Zinc basic salt (ZBS), anionic clay, has attracted great attention as a biocompatible oral drug delivery carrier due to its high anionic exchange capacity and controlled release behavior. In this study, in vitro FAE models were used to observe the transport pathway of 200 and 1000 nm ZBS. In summary, 200 nm ZBS nanoparticles can interact with the epithelial layer and promote intestinal absorption than 1000 nm ZBS particles via transcellular and paracellular pathways without any toxicity, also confirmed by the TEER values and the confocal images of the coculture cell monolayer. Therefore, 200 nm ZBS nanoparticles could be suggested as a promising oral drug delivery carrier.

## Supplementary Material



## Notes

(Figure S1) (A) PXRD patterns of ZBS at (a) 4 °C, (b) 20 °C, (c) 40 °C, and (d) 60 °C of reaction temperature and (e) ZnO, (B) SEM images of ZBS at 4, 20, 40, and 60 °C of reaction temperature and yellow box is ZnO particle; (Figure S2) (A) PXRD patterns of ZBS at (a) 25%, (b) 50%, (c) 75%, (d) 90%, and (e) 100% of ethanol concentration, (B) SEM images of ZBS at 25, 50, 75, 90, and 100% of ethanol concentration; (Figure S3) PXRD patterns of (a) 200 nm ZBS and (b) 1000 nm ZBS (black line: pristine ZBS, red line: ZBS-APS, blue line: ZBS-APS-FITC); (Figure S4) FT-IR spectra of (a) 200 nm ZBS and (b) 1000 nm ZBS (black line: pristine ZBS, red line: ZBS-APS, blue line: ZBS-APS-FITC, green line: FITC); (Figure S5) UV/vis spectra of (a) FITC and (b) ZBS-APS-FITC; (Figure S6) cellular uptake of ZBS-APS-FITC hybrids of varying sizes by target cells. FITC intensity was measured to evaluate intracellular fluorescence after incubation with control (untreated), 200 nm, and 1000 nm ZBS-APS-FITC particles; and zeta potentials and chemical formulas of nanoparticles (PDF)
