# Supplementary material for: Transport Mechanisms of 2D Nanoparticles across a Human Follicle-Associated Epithelium Model
Source: ACS Omega. 2025 Aug 29;10(36):40901–12. doi: 10.1021/acsomega.5c01703 (PMC12444519; doi:10.1021/acsomega.5c01703)
Supplement: Supplementary file 1 [file ao5c01703_si_001.pdf]

Supplementary information

Transport Mechanisms of 2D Nanoparticles across a Human Follicle-Associated Epithelium Model

Sanoj Rejinold N,[†,#] Ji-Yeong Kim,[‖,#] Geun-woo Jin,[⊥] Goeun Choi,[†,∇] Jin-Ho Choy[*†,‡]

[†]Intelligent Nanohybrid Materials Laboratory (INML), Department of Chemistry, College of Science and Technology, Dankook University, Cheonan 31116, Republic of Korea
[‖]Department of Chemistry and Nano Science, Ewha Womans University, Seoul 03760, Republic of Korea
[⊥]R&D Center, Hyundai Bioscience Co., Ltd., Seoul 07990, Republic of Korea
[∇] Department of Nanobiomedical Science, Dankook University, Cheonan 31116, Republic of Korea.
[‡] Division of Natural Sciences, The National Academy of Sciences, Seoul 06579, Republic of Korea

** Correspondence:* Email: jhchoy@dankook.ac.kr

# These Authors contributed equally to this work

Supplementary information

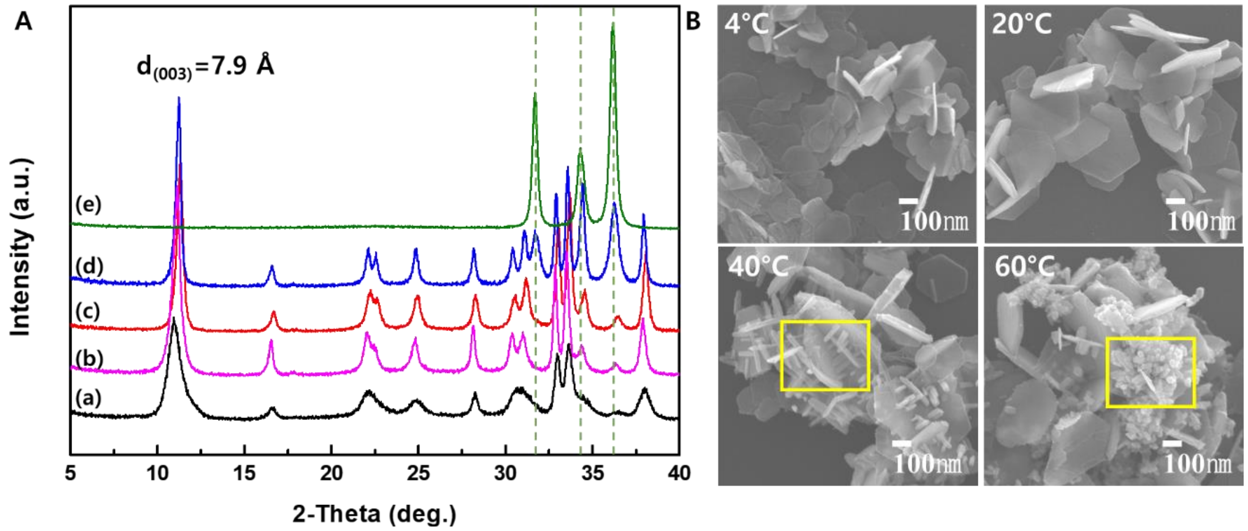

Figure S1. A. PXRD patterns of ZBS at (a) 4°C, (b) 20°C, (c) 40°C and (d) 60°C of reaction temperature and (e) ZnO. B. SEM images of ZBS at 4°C, 20°C, 40°C and 60°C of reaction temperature and yellow box is ZnO particle.

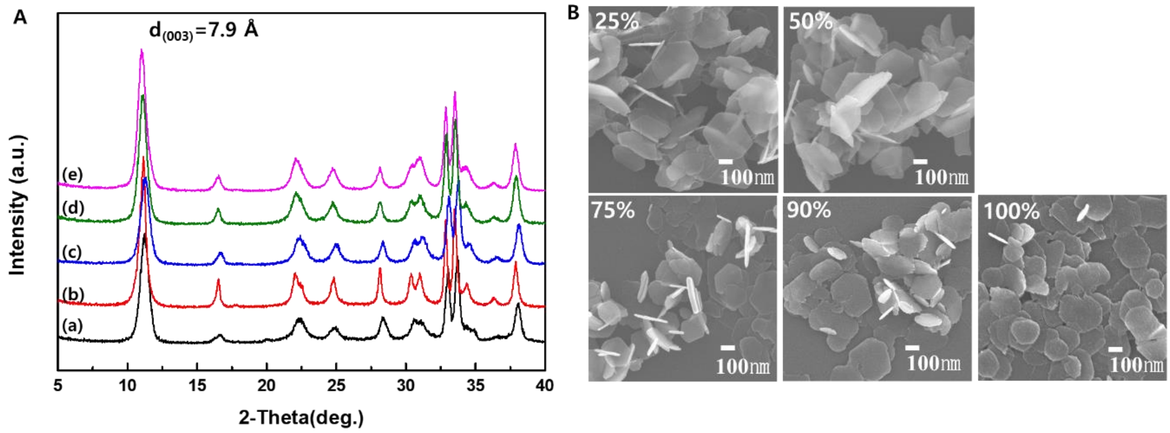

Figure S2. A. PXRD patterns of ZBS at (a) 25%, (b) 50%, (c) 75%, (d) 90% and (e) 100% of ethanol concentration. B. SEM images of ZBS at 25%, 50%, 75%, 90% and 100% of ethanol concentration.

Supplementary information

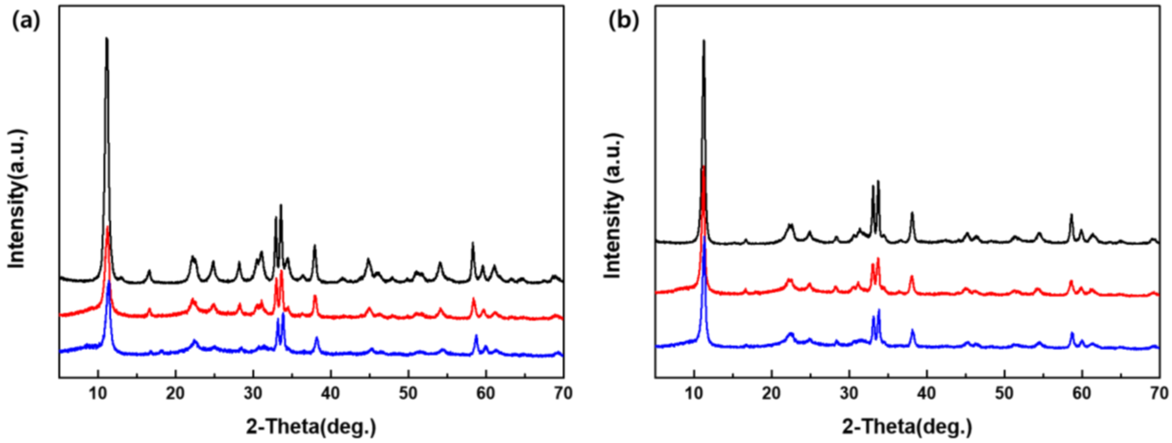

Figure S3. PXRD patterns of (a) 200 nm ZBS and (b) 1000 nm ZBS. (Black line: pristine ZBS, Red line: ZBS-APS, Blue line: ZBS-APS-FITC)

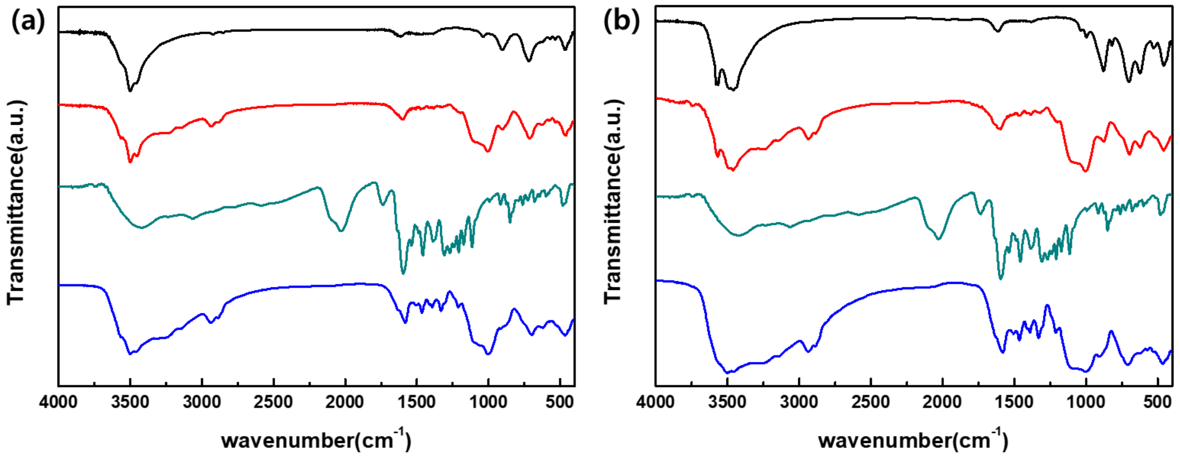

Figure S4. FT-IR spectra of (a) 200 nm ZBS and (b) 1000 nm ZBS. Black line: pristine ZBS, Red line: ZBS-APS, Blue line: ZBS-APS-FITC,Green line : FITC)

For ZBS-APS, the overall spectra were shown and the characteristic band at 3250 and 3100 cm-$^{-1}$ corresponding to $v(NH_2)$ and around 2850 cm^{-1} peak is assigned to $v(CH_2)$. Also, the peak at 830, 700 and 462 cm^{-1} are characteristic of Si-O-Zn bonds.

Supplementary information

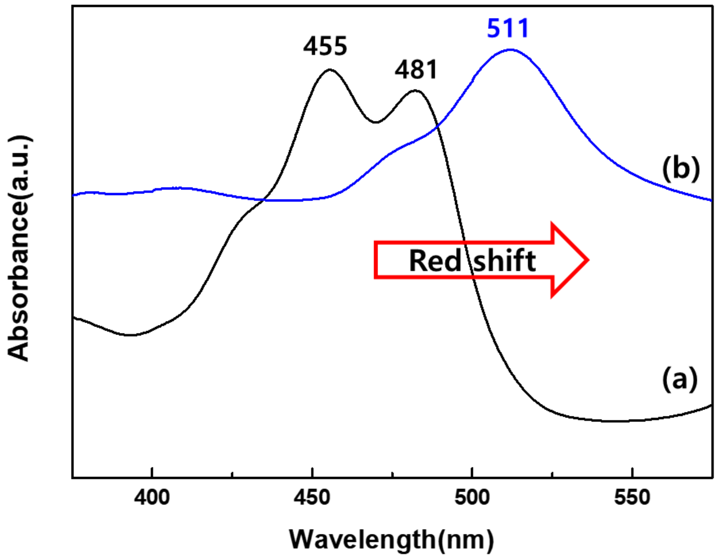

Figure S5. UV/vis spectra of (a) FITC, (b) ZBS-APS-FITC

The original absorption peaks of free FITC were detected at 455 and 481 nm, but after conjugation on ZBS surface, the peaks shifted to 511 nm, respectively shown in Figure S5. Negatively charged ZBS layer can play a role as large electron-releasing group. Therefore, electron releasing layer can provide more electrons on HOMO of FITC, then the absorption of FITC can occur at low energy region. The clear red-shift in UV-vis absorption spectra indicate the conjugation of FITC molecules on ZBS surface.

Supplementary information

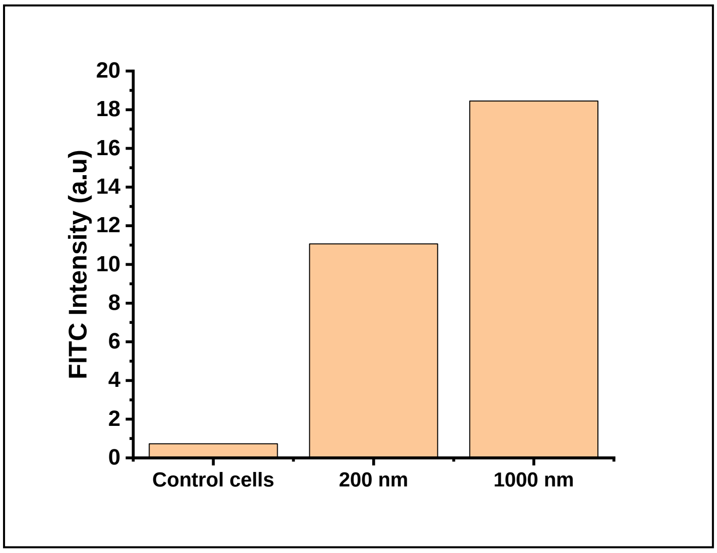

Figure S6. Cellular uptake of ZBS-APS-FITC hybrids of varying sizes by target cells. FITC intensity was measured to evaluate intracellular fluorescence after incubation with control (untreated), 200 nm, and 1000 nm ZBS-APS-FITC particles. A significant increase in fluorescence was observed for both nanoparticle-treated groups compared to control cells, with the 1000 nm particles showing the highest uptake. Data represents FITC intensity in arbitrary units (a.u), indicating size-dependent cellular internalization

Table S1. Zeta-potentials and Chemical formulas of nanoparticles.

	Zeta-potential	Chemical formula [a)]
200 nm ZBS	19.4 ± 1.5 mV	$Zn_5(OH)_8Cl_2$•$0.9H_2O$
200 nm ZBS-ASP-FITC	20.1 ± 1.7 mV	$Zn_5(OH)_8Cl_2$•$0.9H_2O$ •2.43APS•0.051FITC
1000 nm ZBS	21.2 ± 1.6 mV	$Zn_5(OH)_8Cl_2$•$1.1H_2O$
1000 nm ZBS-APS-FITC	21.8 ± 1.5 mV	$Zn_5(OH)_8Cl_2$•$1.1H_2O$ •2.73APS•0.057FITC

a) Calculated from CHNS and ICP analyses
